# The Bacteriological Quality, Safety, and Antibiogram of* Salmonella* Isolates from Fresh Meat in Retail Shops of Bahir Dar City, Ethiopia

**DOI:** 10.1155/2017/4317202

**Published:** 2017-07-25

**Authors:** Melkamnesh Azage, Mulugeta Kibret

**Affiliations:** Department of Biology, Science College, Bahir Dar University, P.O. Box 79, Bahir Dar, Ethiopia

## Abstract

The habit of raw meat consumption in addition to the poor hygienic standards and lack of knowledge contribute to food-borne diseases outbreaks. The objective of this research was to assess the bacterial quality and safety of fresh meat from retail Bahir Dar City, Ethiopia. A total of 30 fresh meat samples were collected from butcher shops. Standard bacteriological methods were used to isolate and enumerate bacteria. Kirby-Bauer disk diffusion method was used for antimicrobial susceptibility testing of* Salmonella* isolates. The mean counts of AMB, TC, and* S. aureus* were log_10_4.53, 3.97, and 3.88 log_10_cfu/g, respectively.* Salmonella* was isolated from 21 (70%) of the samples.* Salmonella* isolates in this study were highly susceptible to ciprofloxacin, gentamycin, and norfloxacin while they were resistant to erythromycin and tetracycline. High rate of multiple drug resistance was also noticed in* Salmonella* isolates. The microbial loads of meat were above the recommended microbial safety limits. Besides this, the isolation rate of* Salmonella* was high and high levels of drug resistance were documented for* Salmonella* isolates. Measures on handling and appropriate personal hygiene practices of workers in the retail shops are recommended to reduce the change of forborne disease outbreaks.

## 1. Introduction

Meat is consumed by many people worldwide because of its nutritive composition. The protein profile of meat consists of amino acids that have been described as excellent due to the presence of all essential ones required by the body [1]. It is considered to be spoiled when it is unfit for human consumption and subjected to changes by its own enzyme, microbial action, and any other factors [2]. Enteric bacteria species can cause infections in humans when undercooked meat products are consumed [3]. The microbiological quality of meat depends on the physical status of the animal at slaughter, the spread of contamination during slaughter and processing, the temperature, and other conditions of storage and distribution [4]. The need for microbial assessment of fresh meats consumption is emphasized and recommended to reduce possible contamination [5].

In Ethiopia, minced or raw beef consumption is usually used for the preparation of a popular traditional Ethiopian dish known as locally “KITFO” and mostly it is consumed raw or partially cooked. This habit is a potential cause for food-borne illnesses in addition to, the common factors such as overcrowding, poverty, inadequate sanitary conditions, and poor general hygiene [6]. The microbiological quality of meat and meat products is very important with regard to public health significance. There are several reports on outbreaks of food-borne illnesses because of consumption of meat [6, 7]. Moreover, antibiotic resistance levels are also elevated among food-borne pathogens such as* Salmonella*,* E. coli*, and* Shigella* [8–10].

The absence of organized slaughter house facility and the existence of small retail outlets have been the two biggest hurdles for hygienic production of meat [11]. It is essential to generate information about the quality of fresh beef sold in retail shops. Hence the present research work was undertaken to determine the bacteriological quality of the meat and determine the antimicrobial susceptibility profiles of* Salmonella* isolates from retail shops of Bahir Dar City, Ethiopia.

## 2. Materials and Methods

### 2.1. Description of the Study Area

This study was conducted in May 2015 in Bahir Dar town, which is the capital of Amhara National Regional State (ANRS) situated in the northern part of Ethiopia. Bahir Dar is located at 11°36′N latitude and 37°23′E longitude and has an elevation of 1840 m above sea level. The area of the town is about 160 km^2^ and there are around 256,999 people living in there [12].

### 2.2. Sample Collection and Bacteriological Analysis

A cross-sectional study was conducted in retail meat shops to determine bacteriological quality and antibiogram of* Salmonella*. A total of 30 retail cut meat samples were collected from 30 purposively selected retail houses between 7:00 and 9:00 am. One kilogram of cut meat was aseptically collected with sterile glove and placed in a sterile glass beaker covered with aluminum foil. The samples were transported to the laboratory in ice box and bacteriological analysis was done within two hours of collection at Food Microbiology Laboratory of School of Chemical and Food Engineering, Bahir Dar University. The ambient temperature at the time of sample collection was 20°C.

Twenty-five grams of meat sample was mixed with 225 ml of 0.1% buffered peptone water (Merck, Darmstadt) and homogenized for 2 minutes by using stomacher (Seward Ltd., UK) [13]. Tenfold serial dilutions (10^−2^–10^−4^) were made from the homogenized sample and 1 ml from each sample of each dilution was taken and used for enumeration of aerobic mesophilic bacteria (AMB), total coliforms (TC), and* S. aureus* and the remaining homogenate was used for the isolation of* Salmonella*.

Enumeration of aerobic mesophilic bacteria was done using the pour plate techniques on plate count agar (Oxoid, England). One ml of homogenized sample was inoculated onto plate count agar, in triplicate and the plates were incubated aerobically at 32°C for a maximum of 48 hrs. After incubation, the plates having 30–300 colonies were counted using colony counter. Uninoculated media were incubated as negative control to check for sterility [14]. Violate Red Bile Agar (VRBA) (Oxoid, England) was used to count total coliforms after incubation at 30–37°C for 24–48 hrs, by using pour plate technique. All purplish red colonies were counted as coliforms [15]. For* Staphylococcus aureus* count, samples were spread-plated in triplicate plates of Mannitol Salt Agar (Oxoid, England) and incubated at 30–37°C for 48 hrs and yellow colonies were counted [14, 15].

### 2.3. Isolation of* Salmonella*

The homogenized sample was incubated at 37°C for 24 hrs and 1 ml of culture was transferred to 10 ml of selenite cysteine broth (SCB) (Himedia, India) and incubated further at 37°C for 24 hrs. A loop full of culture from selenite cysteine broth culture was subcultured onto Xylose lysine deoxycholate (XLD) agar (Oxoid, England) plate and incubated aerobically at 37°C for 24 hrs. After incubation, 2-3 characteristic colonies of* Salmonella* (red colonies with or without black center) were picked and stored on Tryptic Soya Agar (TSA) slants for further purification and used for biochemical characterization and antimicrobial susceptibility tests [16].

### 2.4. Antimicrobial Susceptibility Testing of* Salmonella* Isolates

In vitro antimicrobial susceptibility tests were performed on Mueller-Hinton agar (Oxoid, UK) using Kirby-Bauer disk disc diffusion technique [17]. The antimicrobials tested were ciprofloxacin (CIP, 5 *μ*g), norfloxacin (NOR, 10 *μ*g), amoxicillin (AMC, 30 *μ*g), ampicillin (AMP, 10 *μ*g), chloramphenicol (C, 30 *μ*g), erythromycin (E, 15 *μ*g), gentamicin (CN, 10 *μ*g), nalidixic acid (NA, 30 *μ*g), trimethoprim-sulfamethoxazole (SXT, 25 *μ*g), cefoxitin (FOX, 30 *μ*g), and tetracycline (TE, 30 *μ*g) (Oxoid, UK). Morphologically identical 4–6 bacterial colonies from overnight culture were suspended in 5 ml nutrient broth and incubated for 4 hrs at 37°C. Turbidity of the broth culture was equilibrated to match 0.5 McFarland standards. The surface of Mueller-Hinton agar plate was evenly inoculated with the culture using a sterile cotton swab. The antibiotic discs were applied to the surface of the inoculated agar. After 18–24 hrs of incubation, the diameter of growth inhibition around the discs was measured and interpreted as sensitive, intermediate, or resistant according to Clinical and Laboratory Standards Institute [18]. Reference strain of* E. coli* ATCC 25922 was used as quality control for antimicrobial susceptibility tests.

### 2.5. Data Analysis

Data were analyzed using the statistical package for Social Science (SPSS) version 20 software by descriptive statistics. Results of bacterial counts were expressed in terms of mean log cfu/g and compared with Gulf Standards, 2002 [19] (Table 1). The isolation rate of* Salmonella* prevalence and antimicrobial susceptibility tests were expressed in terms of percentage.

## 3. Results and Discussion

In this study, the aerobic mesophilic bacteria counted in fresh meat ranged between 1.91 and 6.70 log_10_cfu/g having a mean value of 4.53 log_10_cfu/g (Table 2). All 30 samples of fresh meat had high counts of aerobic mesophilic bacteria. In the current study, the total coliform counts detected ranged between 1.40 and 6.50 log_10_cfu/g having a mean value of 3.97 log_10_cfu/g. The mean count of* S. aureus* in fresh meat in this study was 3.88 log_10_cfu/g and ranged between 1.42 and 8.47 log_10_cfu/g (Table 2).

Aerobic mesophilic count is one of the microbiological indicators for food quality and the presence of aerobic organisms reflects existence of favorable conditions for the multiplication of microorganisms [20]. Coliforms are indicators of water or food quality and their presence may be an indication of unhygienic condition [21]. The highest number of* S. aureus* on meat indicates the presence of cross-contamination, which usually related to human skin, hair, hand and discharge from nose, and clothing. High contamination of food with* S. aureus* has been related to improper personal hygiene of employees during handling and processing [12].

The results of this study are comparable to the findings of previous works [22–24]. Other researchers have reported higher and lower aerobic mesophilic, coliform, and* S. aureus* counts [25–27]. The differences might be as a result of differences in study areas, temperature, and personal hygiene practices of the vendors.

The total aerobic counts far exceed the prescribed microbiological safety limits of Gulf Standards [19]. The implication of the findings is that the product is not safe for human consumption, since the samples had counts of aerobic counts exceeding the acceptable limits [28]. In general most of the raw meats sold at butcher shops in this study were potentially hazardous for health.

Among 30 meat samples tested, 21 (70%) were positive for* Salmonella*.* Salmonella* isolates exhibited high level of resistance to erythromycin and tetracycline. The isolates were sensitive to ciprofloxacin, gentamycin, and norfloxacin. There were also intermediate levels of resistances to cefoxitin, ampicillin, and nalidixic acid (Table 3). Among 21 isolates of* Salmonella,* 15 (71.43%) were resistant to two or more antibiotics. Five of the isolates were resistant to three or more antibiotics (Table 4).

With regard to the antimicrobial susceptibility profiles of* Salmonella* isolates, all the* Salmonella* isolates showed high level of sensitivity (95–100%) to gentamycin, ciprofloxacin, norfloxacin, and chloramphenicol while high levels of resistance (66–90%) were documented against erythromycin and tetracycline. From a study done in Ethiopia, Reda et al. [29] and Farzana et al. [30] reported comparable levels of sensitivity and resistance^.^ This could be due to the fact that ciprofloxacin and norfloxacin are relatively expensive and newly introduced, compared to the other common antibiotics. The routine practice of giving antimicrobial agents to domestic livestock as a means of preventing and treating diseases, as well as promoting growth, is an important factor in the emergence of antibiotic-resistant bacteria that are subsequently transferred to humans through the food chain [31, 32]. Most infections with antimicrobial-resistant* Salmonella* are acquired by eating contaminated foods of animal origin [33, 34].

## 4. Conclusion

This study revealed high level of contamination in fresh meat as indicated by high aerobic mesophilic,* S. aureus,* and coliform counts which are above the recommended microbial safety limits. High bacterial loads and isolation of drug resistant* Salmonella* suggest a potential health risk to the consumers from the consumption of raw meat. These indicate poor handling and personal hygiene practices of workers in the retail shops and risk of food-borne disease. Investigation on antibiotic use in animal and animal feed is recommended.

## Figures and Tables

**Table 1:** Guideline levels for determining microbial quality of ready-to-eat food (Gulf Standards and NSW Food Authority).

Microbial groups	Good	Acceptable	Unsatisfactory	Unacceptable and potentially dangerous
Aerobic mesophilic count	<10^4^	10^4^–<10^6^	≥10^6^	N/A
Total coliform count	<10^2^	10^2^–10^4^	≥10^4^	N/A
*S. aureus *count	<10^2^	10^2^–10^3^	10^3^–<10^4^	≥10^4^
Pathogens	Not detected in 25 g of	—	—	Detected in 25 g of

**Table 2:** Bacterial counts of fresh meat in Bahir Dar town, May, 2015.

Bacterial counts	Minimum count (log_10_cfu/g)	Maximum count (log_10_cfu/g)	Mean ± SD (log_10_cfu/g)
AMC	1.91	6.70	4.53 ± 1.24
TC	1.40	6.50	3.97 ± 1.42
*S. aureus*	1.42	8.47	3.88 ± 1.81

**Table 3:** Antibiotic susceptibility pattern of *Salmonella* isolates in Bahir Dar town, May, 2015.

Antimicrobial Agents	Resistant No (%)	Intermediate No (%)	Sensitive No (%)
Ciprofloxacin	0 (0)	0 (0)	21 (100)
Nalidixic acid	0 (0)	2 (9.5)	19 (90.5)
Erythromycin	19 (90.5)	2 (9.5)	0 (0)
Ampicillin	5 (23.8)	4 (19)	12 (57.2)
Tetracycline	14 (66.7)	0 (0)	7 (33.3)
Trimethoprim-sulfamethoxazole	2 (9.5)	0 (0)	19 (90.5)
Gentamycin	0 (0)	0 (0)	21 (100)
Cefoxitin	2 (9.5)	7 (33.3)	12 (57.2)
Amoxicillin	2 (9.5)	2 (9.5)	17 (81)
Chloramphenicol	0 (0)	1 (4.8)	20 (95.2)
Norfloxacin	0 (0)	0 (0)	21 (100)

**Table 4:** MDR pattern of *salmonella* isolates in Bahir Dar town, June, 2015.

Resistance pattern	*Salmonella* isolates No (%)
Resistant to two antibiotics	
E-TE	8 (38)
E- SXT	1 (4.8)
TE-AMP	1 (4.8)
Resistant to three antibiotics	
E-TE-AMP	2 (9.5)
Resistant to four antibiotics	
E-TE-AMP-AMC	1 (4.8)
E-TE-AMP- SXT	1 (4.8)
E-TE-FOX-AMC	1 (4.8)
